# Regenerative defect in *vastus lateralis* muscle of patients with chronic obstructive pulmonary disease

**DOI:** 10.1186/1465-9921-15-35

**Published:** 2014-03-25

**Authors:** Marie-Eve Thériault, Marie-Ève Paré, Bruno B Lemire, François Maltais, Richard Debigaré

**Affiliations:** 1 Centre de recherche Institut universitaire de cardiologie et de pneumologie de Québec, Université Laval, Québec, QC G1V 4G5, Canada

**Keywords:** Satellite cells, COPD, Muscle, Regeneration, Atrophy

## Abstract

**Background:**

Impaired skeletal muscle regeneration could contribute to the progression of muscle atrophy in patients with chronic obstructive pulmonary disease (COPD).

**Methods:**

Satellite cells and myogenesis-related proteins were compared between healthy subjects and patients with COPD, with or without muscle atrophy. Satellite cells were isolated and cultured to assess their proliferative and differentiation aptitudes.

**Results:**

Although satellite cell numbers in muscle samples were similar between groups, the proportion of muscle fibers with central nuclei was increased in COPD. In muscle homogenates, increased expression of MyoD and decreased expression of myogenin and MRF4 were observed in COPD. In cultured satellite cells of patients with COPD, increased protein content was observed for Pax7, Myf5 (proliferation phase) and myogenin (differentiation phase) while myosin heavy chain protein content was significantly lower during differentiation.

**Conclusion:**

In COPD, the number of central nuclei was increased in muscle fibers suggesting a greater number of attempts to regenerate muscle tissue than in healthy subjects. Myogenesis signaling was also altered in muscle homogenates in patients with COPD and there was a profound reduction in the differentiation potential in this population as indicated by a reduced ability to incorporate myosin heavy chain into newly formed myotubes. Collectively, these results indicate that skeletal muscle regenerative capacity termination is impaired in COPD and could contribute to the progression of muscle atrophy progression in this population.

## Background

Skeletal muscle atrophy is observed in a variety of acute and chronic conditions [1-5] including chronic obstructive pulmonary disease (COPD) [6]. In COPD, this loss of muscle mass has a significant impact on quality of life [7] and is associated with premature death [8]. Many biochemical factors have been proposed to initiate and promote the development of skeletal muscle atrophy in COPD [9]. An impaired capacity for muscle regeneration has been hypothesized to take part in this process [10]. However, no studies have yet investigated whether the ability of satellite cells to proliferate and differentiate could be involved in the atrophying process in the context of COPD.

Skeletal muscle possesses a remarkable capacity to regenerate itself, which largely depends on muscle satellite cells and the expression of myogenic regulatory factors (MRFs). Upon activation [11-13], satellite cells proliferate into myoblasts and migrate to the requested site. In the activated state, these cells will enter the cell cycle through the expression of early MRFs such as MyoD and Myf5 [14]. Eventually, newly formed myoblasts will exit the cell cycle and differentiate into myotubes. At this stage cells will express two distinct MRFs, myogenin and MRF4. Fully differentiated myotubes will fuse to injured myofibers for repair or fuse together to form a new one [15]. To preserve an adequate population of muscle satellite cells during adulthood, some newly formed myoblasts will return to a quiescent state [16,17].

Transition of activated satellite cells to highly proliferative myoblasts and then to differentiated myotubes is a well regulated process. First, up-regulation of Notch signaling promotes the transition of activated satellite cells to highly proliferative myoblasts [18]. A subsequent decline in Notch signaling activity is necessary for differentiation of progenitor cells into fusion competent myoblasts [19]. Increases in the expression of Numb is known to switch off Notch signaling [18]. The transition from Notch to Wnt signaling in myogenic progenitors is a prerequisite step for differentiation [20].

Muscle is highly dynamic and muscle mass maintenance relies on the delicate balance between protein synthesis and degradation as well as the additions and loss of myonuclei. We hypothesized that the regenerative process is impaired in COPD patients presenting muscle atrophy compared to healthy individuals. This hypothesis is supported by our recent finding that satellite cell senescence is likely occurring in lower limb muscle of patients with COPD and low muscle mass [21]. To further test this hypothesis, the research protocol was divided in two distinct aims: 1) in muscle tissue, to evaluate satellite cell number, by counting the occurrence of central nuclei (a marker of newly fused satellite cells) and to study the expression pattern of myogenesis-related proteins; and 2) in isolated and cultured satellite cells, to assess proliferation, MRFs expression and differentiation as key steps of the regeneration process. This study confirms that although limb muscle of patients with COPD and healthy age matched controls contains the same number of satellite cells, their myogenic capacities are impaired in COPD.

## Methods

### Subjects

Seventeen males with Global Initiative for Chronic Obstructive Lung Disease (GOLD) stage III and IV disease and nine healthy male subjects with normal lung function were consecutively recruited for this study. None of them were part of a previous study on satellite cell biology in COPD^21^. COPD diagnosis was based on a past smoking history (> 10 pack-year) and pulmonary function tests showing persistent airflow obstruction (forced expiratory volume in 1 s [FEV_1_] < 50% of predicted value and FEV_1_/forced vital capacity [FVC] < 70%) [22]. All patients with COPD were in a stable condition at the time of the study and were neither suffering from any other diseases nor using oral corticosteroids. The institutional ethics committee (Comité d’éthique, Institut Universitaire de cardiologie et de pneumologie de Québec) approved the study protocol and each patient signed a written informed consent form.

### Study design

To address aim #1, muscle sections and homogenates of all study participants were used respectively for immunostaining and protein measurements. Eight patients with COPD and seven healthy subjects had sufficient amount of muscle tissue for isolation and culture of satellite cells and fulfilled the second aim of this study.

### Pulmonary function, anthropometric measurements and body composition

Standard pulmonary function tests including spirometry, lung volumes, and carbon dioxide diffusion capacity were obtained in all subjects during the initial evaluation according to previously described guidelines [23]. Results were related to previously published normal values [24]. Height and weight were measured according to standardized methods [1]. Mid-thigh muscle cross-sectional area (MTCSA) was determined using computed tomography, as previously described [8].

### Muscle biopsy

One needle biopsy of the *vastus lateralis*, performed as described by Bergström and routinely done in our laboratory, [25] was obtained from each participant. Muscle specimens were divided in two parts. One was frozen in liquid nitrogen and stored at -80°C for analyses on immunostained muscle sections and on muscle homogenates (aim #1)). When sufficient tissue was available, the second part of the muscle specimen (COPD n = 8; controls n = 7) was placed in a sterile plate filled with phosphate-buffered saline (PBS), covered and transported on ice under sterile cell culture hood to perform satellite cell isolation (aim #2).

### Fiber typing

Muscle sections were stained with a monoclonal anti-myosin (skeletal, fast) antibody (Sigma-Aldrich, St-Louis, MO, USA) to detect type II muscle fibers using a standard immunohistochemistry protocol. The proportion of type I (nonstained), and II (darkly stained) fibers was assessed and calculated as the number of fibers of each type divided by the total number of muscle fibers on the cryosection.

### Satellite cells and myonuclei staining

To assess the number of satellite cells in muscle tissue sections, immunostaining against transcription factor Pax7 (R&D Systems) was performed. Laminin was used to delineate muscle fibers. Nuclei were labeled using DAPI. Nuclei located deep to the basal lamina and positive for Pax7 were counted as satellite cells. Satellite cells were counted in over 100 myofibers and reported as a ratio. Muscle sections (10 μm) were fixed with ice-cold acetone and methanol 60/40 (v/v) at – 20°C for 20 minutes, washed with PBS, incubated with a blocking solution (horse serum 1%) for 1 hour and then incubated with the primary antibody overnight at 4°C in a humidified chamber. The primary antibody Pax-7 (R&D Systems) and laminin (Dako, Glostrup, Denmark) excess was cleaned with PBS, incubated with their specific secondary antibodies, a goat anti-mouse Alexa Fluor® 488 (R&D Systems) and a goat anti-rabbit Alexa Fluor® 546 (Invitrogen Corporation, Carlsbad, CA, USA) for 1 hour, washed with PBS and then incubated with DAPI for 15 minutes. Slides were analyzed and images were captured using a Nikon Eclipse E600 microscope (Nikon Corporation, Tokyo, Japan). Immunofluorescences were performed in duplicate on two different muscle cryosections.

### Satellite cell isolation and culture

Satellite cells were isolated and cultured as previously published [26]. Briefly, fresh muscle samples were placed in a sterile plate filled with phosphate-buffered saline (PBS), covered and transported on ice under sterile cell culture hood. All subsequent manipulations were performed under sterile conditions. Muscle samples were minced into a slurry using a scalpel and subjected to enzymatic digestion for 1 h with 0,33% collagenase (Cedarlane, Hornby, ON, Canada) at 37˚C with gentle agitation. The digested muscle specimens were then dissociated by triturating them several times and filtered through a 100 μm filter (BD Biosciences, Franklin Lakes, NJ, USA). Cells were plated and grown on collagen coated dishes (Sigma-Aldrich) in growth medium made of Dulbecco’s modified Eagle’s medium (DMEM) with 1% glucose (HyClone, Logan, UT, USA) supplemented with 20% fetal bovine serum, penicillin 50 U/ml, streptomycin 50 μg/ml (HyClone) and 5 ng/ml bFGF (Promega, Madison, WI, USA) in a humidified 37°C, 5% CO_2_ incubator. The medium was changed every two days and the cultures examined by inverted-phase microscopy. When cultured cells reached a confluence of 70% they were dissociated enzymatically with trypsin (Gibco, Carlsbad, CA, USA) and seeded for immediate propagation, or frozen into medium containing 10% DMSO for later use. To induce differentiation into myotubes, near to confluence myoblasts were placed in differentiation medium made of DMEM with 1% glucose and supplemented with 2% horse serum (HS), penicillin 50 U/ml, and streptomycin 50 μg/ml (HyClone). Differentiation medium was replaced every day for 7 consecutive days. Images taken from myoblasts and myotubes were captured with a Nikon 950 digital camera (Nikon Corporation). To ensure the exclusivity of the myogenic nature of the cell culture, Pax-7 (R&D Systems, Minneapolis, NE, USA) staining was performed. Immunohistochemistry was performed using Vectastain Elite ABC Kit according to the instructions provided by the manufacturer (Vector Labs, Burlingame, CA, USA).

### Proliferation assay

After the first passage, satellite cells were plated at a density of 5 × 10^4^ cells in a 60 mm cultures dish. Every 24 h, cells were trypsinized and counted using a hemacytometer during four successive days.

### MRFs expression

To measure progression through myogenesis, 2 × 10^5^ cells were placed in a 100 mm dish after their second passage. Cells were allowed 24 hours to settle and adhere before starting the experiment. Near confluent myoblasts were placed in differentiation medium for seven consecutive days. Whole cell lysates were performed every 24 h until complete differentiation for a total of nine days in culture. Western blotting against Pax7 (R&D), MyoD, Myf5, Myogenin, MRF4 (Santa Cruz Biotechnology, Santa Cruz, CA, USA), and Numb (Cell Signaling Technology, Danvers, MA, USA) was performed to quantify myogenesis related protein accumulation.

### Differentiation assay

To measure differentiation rate, 2 × 10^5^ cells were placed in a 60 mm dish after their second passage. Near confluent myoblasts were placed in differentiation medium for seven consecutive days. Cell lysates were prepared every 24 h until seven days of differentiation. To quantify differentiation, western blotting against monoclonal anti-myosin (skeletal, fast) antibody (Sigma-Aldrich) was performed.

### Statistical analysis

Results are expressed as mean (± SEM). Data from patients with COPD and healthy controls were analyzed using a one-way analysis of variance (ANOVA). Because low muscle mass is related to poor quality of life [27], reduced functional capacity [7] and survival [8], immunostaining and tissue homogenate data obtained from patients with COPD were subsequently subdivided according to a MTCSA <70 cm^2^ or >70 cm^2^ for further sub-analysis purposes. The 70 cm^2^ MTCSA threshold was used to define muscle atrophy based on a previous study showing that mortality was significantly increased in patients with MTCSA below this cutoff value [8]. Differences for all variables between patients with COPD with MTCSA <70 cm^2^ and >70 cm^2^ and healthy controls were analyzed using a one-way analysis of variance (ANOVA). Muscle mass was not considered in the analysis of cultured satellite cell data because the composition of the study groups was based on amount of tissue available for cell isolation and not on baseline subject characteristics. Differences were considered to be significant when p < 0.05.

## Results

### Muscle homogenates

#### *Subject’s characteristics*

Anthropometric characteristics and pulmonary function data are provided in Table 1. Patients with COPD with MTCSA > 70 cm^2^ and healthy subjects did not significantly differ in age and body mass index. Patient with COPD and MTCSA < 70 cm^2^ had a significantly lower body mass index compared to the other two groups. On average, patients with COPD had moderate-to-severe airflow obstruction: eight patients had stage III COPD and nine patients had stage IV disease according to the GOLD classification [28]. Type I fiber proportion was significantly decreased in patients with COPD, while type II fiber proportion was reciprocally increased.

**Table 1:** Subject characteristics

**Characteristics**	**Control (n = 9)**	**COPD > 70 cm**^**2 **^**(n = 11)**	**COPD < 70 cm**^**2 **^**(n = 6)**
**Age (years)**	63 ± 2.3	64 ± 2.3	70 ± 2.4
**BMI (kg/m**^**2**^**)**	29.1 ± 2.4	28.1 ± 2.0	21.7 ± 2.6^b^
**FEV**_**1**_**, L**	3.3 ± 0.2	1.0 ± 0.2^a^	0.8 ± 0.1^a^
**FEV**_**1, **_**% predicted**	106.3 ± 4.1	36.0 ± 4.9^a^	26.0 ± 3.8^a^
**FEV**_**1**_**/FVC, %**	76.0 ± 1.6	36.1 ± 1.8^a^	29.0 ± 2.6^b^
**TLC, % predicted**	98.6 ± 6.5	121.6 ± 6.6^a^	125.7 ± 4.8^a^
**RV, % predicted**	90.1 ± 15.3	173.2 ± 22.8^a^	195.5 ± 22.9^a^
**DL**_**CO**_**, % predicted**	96.9 ± 3.2	57.4 ± 6.7^a^	40.0 ± 2.8^b^
**MTCSA (cm**^**2**^**)**	120.3 ± 3.4	86.3 ± 4.2^a^	60.8 ± 3.6^b^
*Type I fiber*			
**Distribution, %**	51.5 ± 5.7	34.7 ± 9.3^a^	34.9 ± 3.5^a^
**CSA, (μm**^**2**^**)**	5170 ± 471	4711 ± 340	4883 ± 607
*Type II fiber*			
**Distribution, %**	44.1 ± 5.7	65.4 ± 9.3^a^	65.2 ± 3.5^a^
**CSA, (μm**^**2**^**)**	4216 ± 358	4359 ± 540	4096 ± 480

Values are means ± SEM.

^a^statistically significant difference from control (p < 0.05).

^b^statistically significant difference from COPD > 70 cm^2^ (p < 0.05).

*Definition of abbreviations*: *BMI* Body mass index, *FEV*_*1*_ Forced expiratory volume in 1 second, *TLC* total lung capacity, *RV* Residual volume, *DLCO* Diffusing capacity of carbon monoxide, *MTCSA* Mid-thigh cross-sectional area, *CSA* Cross-sectional area.

#### *Satellite cells and central nuclei quantification*

**Figure 1:**
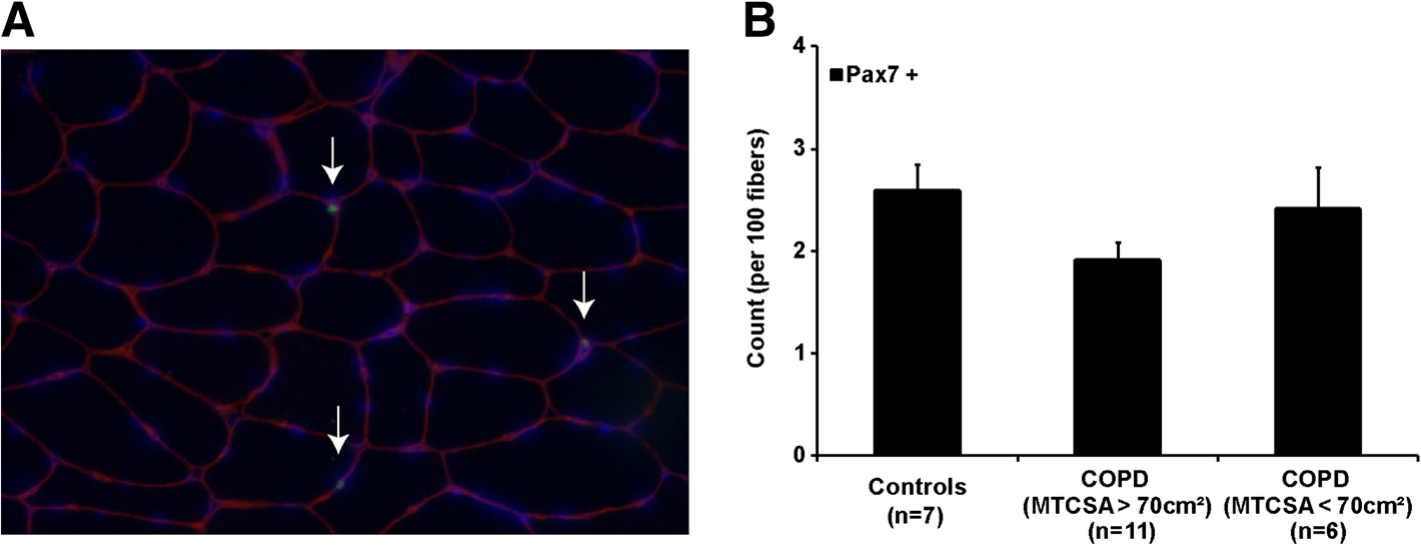
**Satellite cell detection performed by immunostaining. (A)***Vastus lateralis* muscle cryosections were immunostained for co-expression and localization of nuclear Paired box transcription factor 7 (Pax7, green), DAPI (blue) and laminin (red). Labeled nuclei located deep to the basal lamina and positive for pax7 were counted as satellite cells (white arrow). **(B)** Labeled nuclei located deep to the basal lamina and positive for Pax7 were counted as satellite cells. The number of satellite cells is expressed over 100 fibers and reported as a ratio; ANOVA; p > 0.05. Ratios are calculated from 9 healthy subjects, 11 COPD (MTCSA > 70 cm^2^) and 6 COPD (MTCSA < 70 cm^2^).

A representative *vastus lateralis* cryosection immunostained for Pax7 (expressed by satellite cells) and laminin (a major constituent of the basal lamina) is depicted in Figure 1A. The proportion of myofibers positive for Pax7 did not significantly differ between groups (Figure 1B).

**Figure 2:**
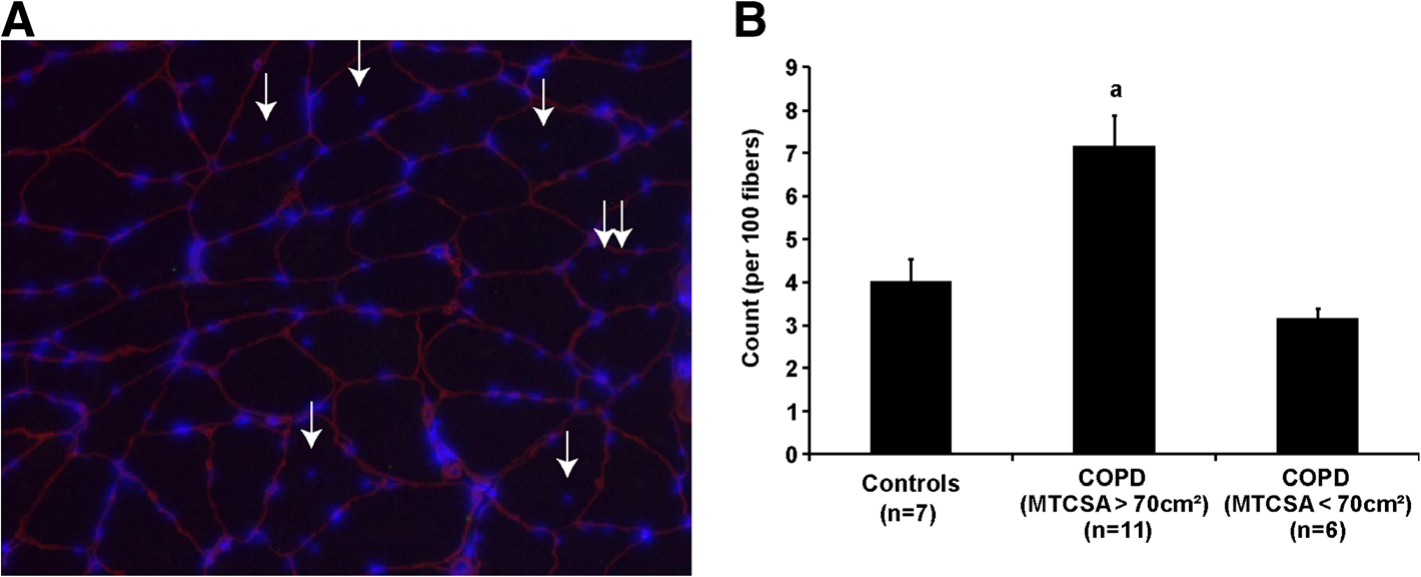
**Central nuclei detection performed by immunostaining. (A)** Cryosections were analyzed by immunostaining for nuclei (blue) and laminin (red). Immuno-detection is shown for 10 μm skeletal muscle cryosections. The number of central nuclei (white arrows) is expressed over 100 fibers and reported as a ratio. Ratios are calculated from 9 healthy subjects, 11 COPD (MTCSA > 70 cm^2^) and 6 COPD (MTCSA < 70 cm^2^). **(B)** Nucleus inside the perimeter of a given fiber delimited by laminin was counted as a central nucleus, expressed over 100 fibers and reported as a ratio. When a is present: statistically significant difference from control. When b is present b: statistically significant difference from COPD > 70 cm^2^ (ANOVA; p < 0.05).

A representative cryosection immunostained for nucleus and laminin is provided in Figure 2A. The number of central nuclei (a marker of newly fused satellite cells) per 100 muscle fibers was significantly higher in patients with COPD and preserved muscle mass (MTCSA > 70 cm^2^) compared to patients with COPD and muscle atrophy (MTCSA < 70 cm^2^) and healthy subjects (7.2 ± 0.7 vs 3.2 ± 0.2 vs 4.0 ± 0.5 respectively; p < 0.0001) (Figure 2B).

#### *Expression of myogenic regulatory factors*

**Figure 3:**
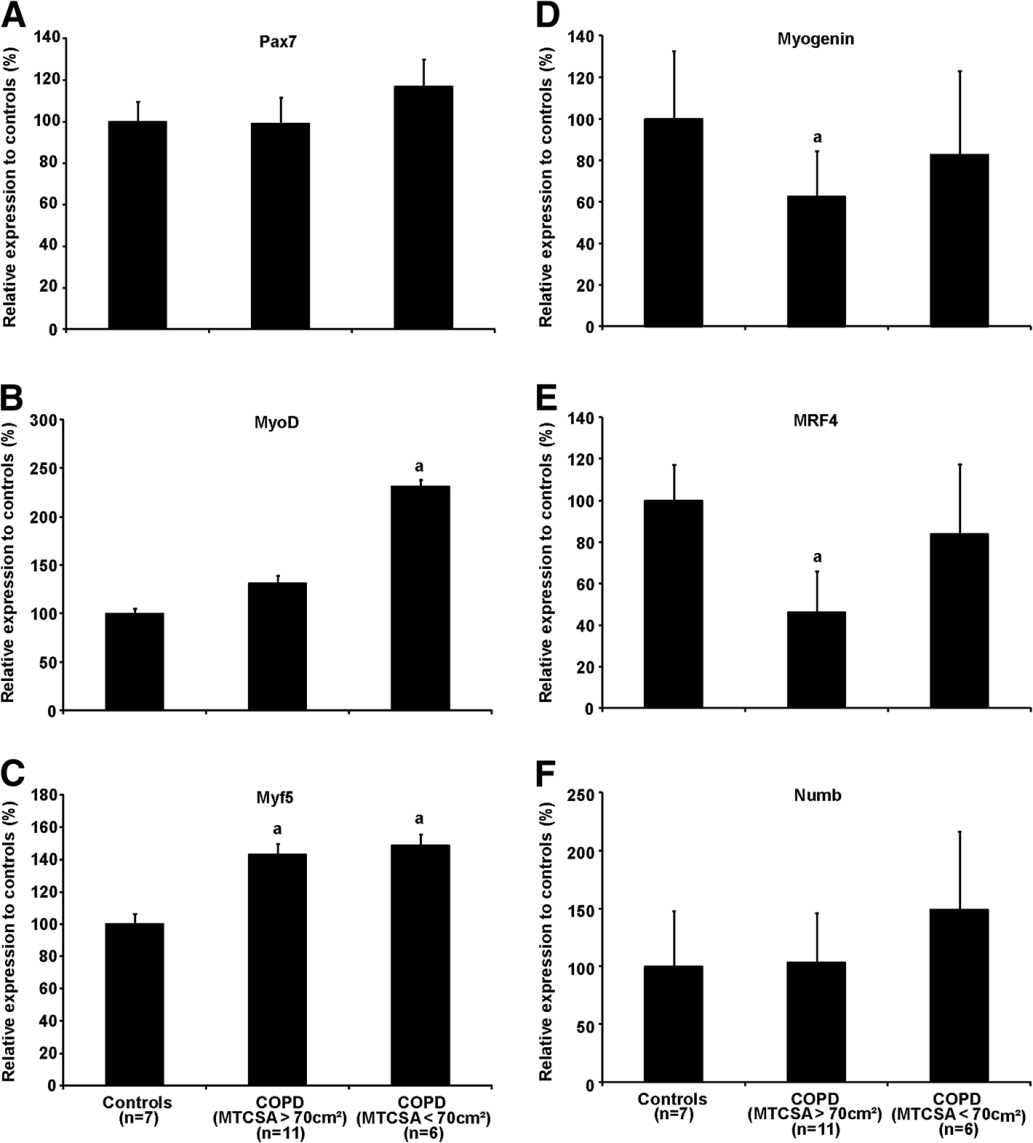
**Western blot against Pax7 (A), MyoD (B), Myf5 (C), Myogenin (D), MRF4 (E) and Numb (F) total protein extract of the muscle biopsies obtained from the *vastus lateralis*.** Results are reported as a percentage compared to controls subjects. When a is present: statistically significant difference from control. When b is present b: statistically significant difference from COPD > 70 cm^2^ (ANOVA; p < 0.05).

A significant increase in MyoD protein content was observed in patients with COPD with MTCSA < 70 cm^2^ (Figure 3) compared to healthy subjects (2.31 fold-increase, p < 0.05) and to patients with COPD with MTCSA > 70 cm^2^ (1.31 fold-increase, p < 0.05). Myf5 protein content was significantly increased in both groups of patients with COPD compared to healthy subjects (1.42 and 1.49 fold-increase respectively, p < 0.05). There was a significant decrease in myogenin (0.62 fold-decrease, p < 0.005) and MRF4 (0.46 fold-decrease, p < 0.005) protein contents in patients with COPD with MTCSA > 70 cm^2^ compared to healthy subjects. There was no difference between Pax7 and Numb (total amount) protein content between all three groups.

### Satellite cell analyses

To further characterize satellite cells present in the *vastus lateralis* biopsies, a protocol to isolate and culture muscle satellite cells was developed. Proliferation and differentiation rates of the satellite cells were determined using this model.

#### *Subject’s characteristics*

Anthropometric characteristics and pulmonary function data for subjects in whom primary muscle cell culture was performed are provided in Table 2.

**Table 2:** Subjects characteristics used for primary culture

**Characteristics**	**Control (n = 7)**	**COPD (n = 8)**
**Age**	63 ± 2.3	66 ± 2.3
**BMI (kg/m**^**2**^**)**	29.1 ± 2.4	28.8 ± 2.0
**FEV**_**1**_**, L**	3.3 ± 0.2	1.0 ± 0.1^a^
**FEV**_**1, **_**% predicted**	106.3 ± 4.1	37.0 ± 4.0^a^
**FEV**_**1**_**/FVC, %**	76 ± 1.6	36.5 ± 1.1^a^
**TLC, % predicted**	98.6 ± 6.5	118.0 ± 5.5
**RV, % predicted**	90.1 ± 15.3	163.5 ± 15.9^a^
**DL**_**CO**_**, % predicted**	96.9 ± 3.2	53.3 ± 5.8^a^
**MTCSA (cm**^**2**^**)**	120.3 ± 3.4	82.3 ± 6.5^a^
**Satellite cells (Pax7+/100 fibers)**	2.6 ± 0.4	1.7 ± 0.3^a^
**Central nuclei (count/100 fibers)**	4.0 ± 0.8	6.5 ± 1.0^a^

Values are means ± SEM.

^a^statistically significant difference from control (p < 0.05).

*Definition of abbreviations*: *BMI* Body mass index, *FEV*_*1*_ Forced expiratory volume in 1 second, *TLC* total lung capacity, *RV* Residual volume, *DLCO* Diffusing capacity of carbon monoxide, *MTCSA* Mid-thigh cross-sectional area.

#### *Isolation and characterization of satellite cells*

Cultured cells demonstrated myoblast-like and myotubes-like shapes in growing and differentiation states. Positive staining for Pax7 confirmed the satellite cell phenotype of these cells. In all cultures, at least 95% of freshly isolated muscle progenitor cells were classified as Pax7+ satellite cells by immunohistochemistry. Cells were grown under sterile conditions and no signs of any contamination were observed.

When satellite cells were isolated from the muscle samples and put into culture, a delay in their adhesion into dishes was observed. Satellite cells isolated from patients with COPD took 7.3 ± 0.3 days to adhere and proliferate compared to 5.5 ± 0.3 days (p < 0.05) for those isolated from healthy subjects.

#### *Proliferation*

**Figure 4:**
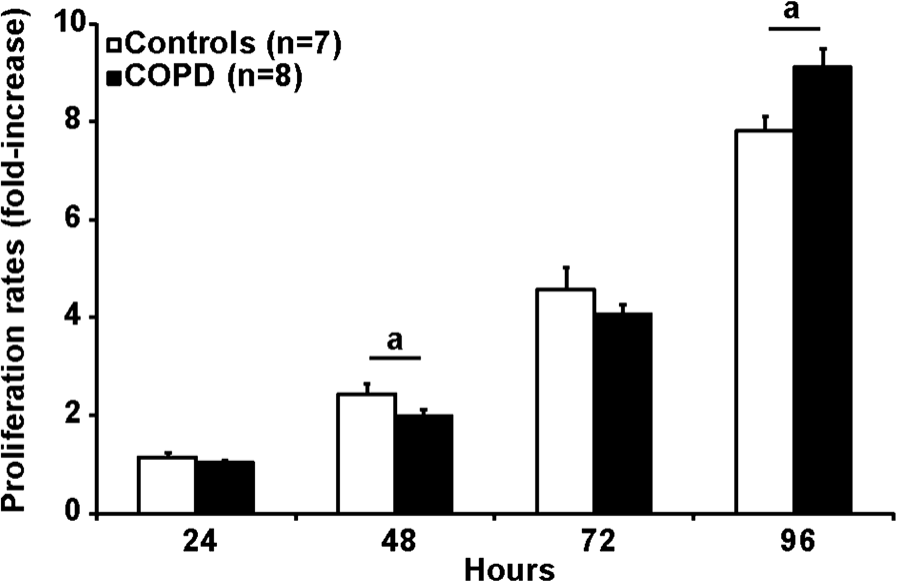
**Proliferative potential in satellite cells isolated and cultured from healthy subjects (white bars) and patients with COPD (black bars).** Following the first passage, 5 × 10^4^ cells were seeded on a 60 mm-dish and were counted every next 24 hours during 96 consecutive hours. Values are expressed as mean ± SEM. When a is present: statistically significant difference from control (ANOVA; p < 0.05).

Cell proliferation was evaluated by counting growing cells every 24 hours over a 96-hour period. The number of cells at each time point is presented in Figure 4. A slight but statistically significant decrease in cell proliferation was observed at 48 hours in patients with COPD compared to healthy subjects (p < 0.01). At 72 hour, proliferation rate in COPD was similar compared to healthy subjects with even a significant increase in proliferation at 96 hours in COPD compared to healthy individuals (p < 0.005).

#### *Myogenesis*

**Figure 5:**
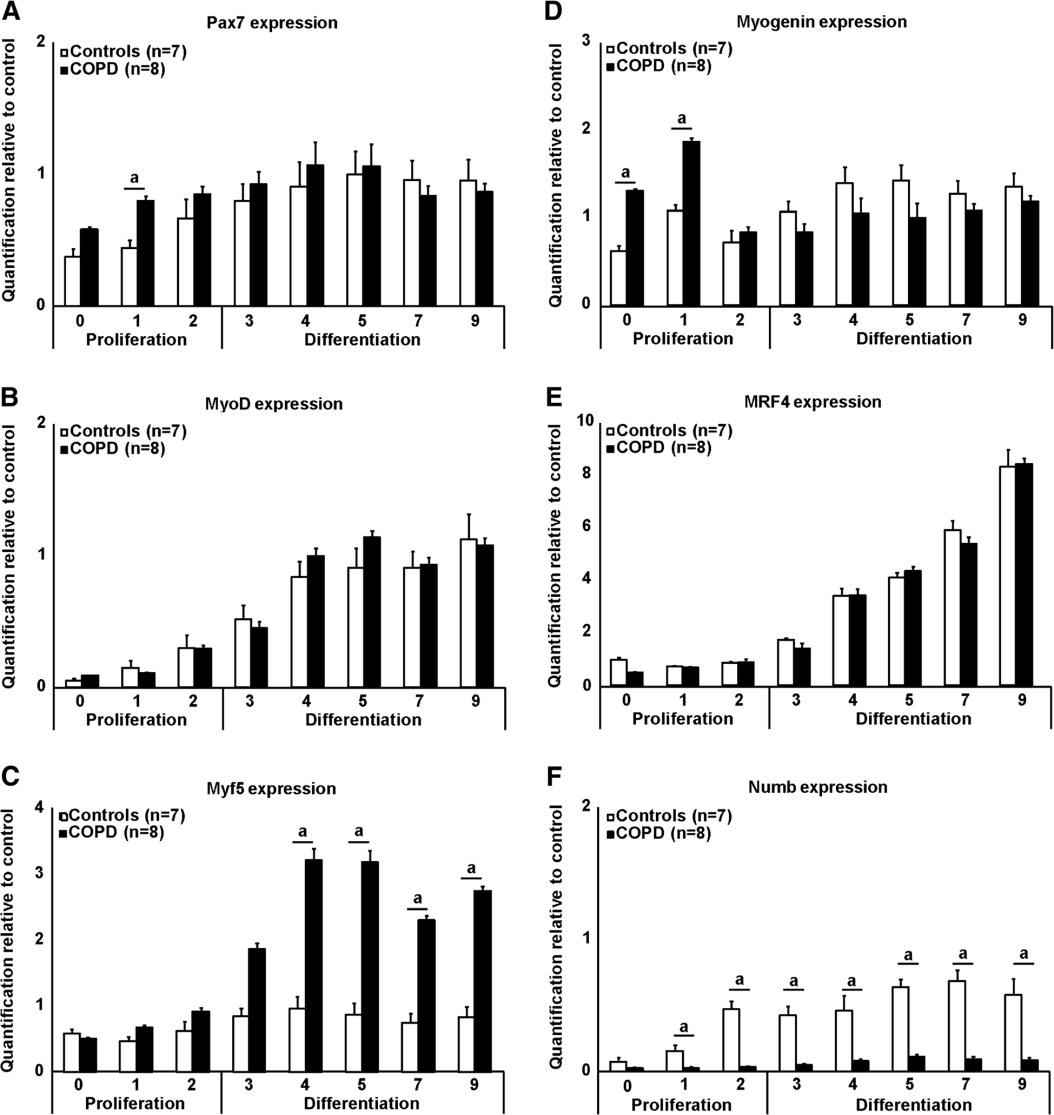
**A) Pax7, B) MyoD, C) Myf5, D) Myogenin, E) MRF4 and F) Numb protein accumulation during myogenesis in satellite cells isolated and cultured from healthy subjects (white bars; n = 7) and patients with COPD (black bars; n = 8).** Protein extraction was performed daily, the first three days represent the myoblasts phase past than is the differentiation phase (day 3 to day 9). Signal analysis was calculated by densitometry and reported over a positive control. When a is present: statistically significant difference from control (ANOVA; p < 0.05).

Cells were grown in proliferation medium for two days than placed in differentiation medium for the remaining period of time. In COPD, there was a significant increase in Pax7 accumulation after one day in growing culture medium compared to healthy subjects (2.0 fold-increase, p < 0.05) (Figure 5A). MyoD protein accumulation during this period was similar between groups while significant increases in Myf5 protein accumulation (p < 0.05) were observed in COPD compared to healthy subjects in our distinct days during differentiation (days 4 (3.2 fold-increase), 5 (3.6 fold-increase), 7 (2.9 fold-increase) and 9 (3.5 fold-increase) (p < 0.05)) (Figure 5B and C, respectively). Significant increases in the accumulation of myogenin in patients with COPD compared to healthy subjects were observed at days 0 (2.2 fold-increase) and 1 during proliferation (1.7 fold-increase) (p < 0.05) (Figure 5D). The accumulation of MRF4 did not differ between groups (Figure 5E). Significant decreases in the accumulation of Numb in patients with COPD compared to healthy subjects were observed throughout the proliferation and differentiation periods (Figure 5F).

#### *Differentiation*

**Figure 6:**
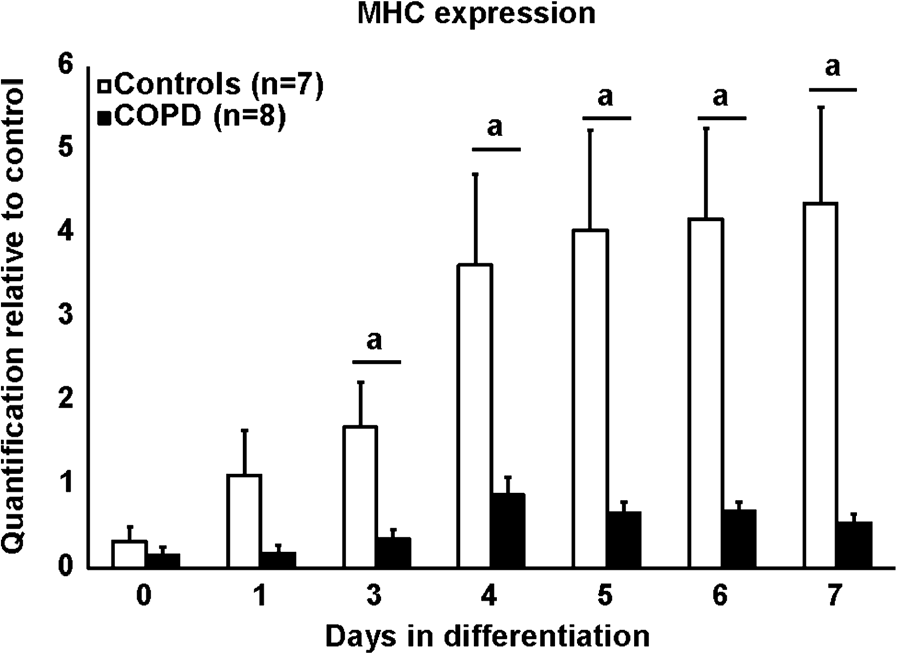
**Skeletal myosin fast twitch protein accumulation during myogenesis in satellite cells isolated and cultured from healthy subjects (white bars; n = 9) and patients with COPD (black bars; n = 8).** Protein extraction was performed daily after initial differentiation. Signal analysis was calculated by densitometry and reported over a positive control. When a is present: statistically significant difference from control (ANOVA; p < 0.05).

At the end of the myogenic process, the capacity of myoblasts to fully differentiate into myotubes was determined by quantifying the accumulation of MHC over a period of seven days (Figure 6). After three days of differentiation, there was a profound decrease in the accumulation of MHC in COPD compared to healthy subjects that persisted throughout the rest of the differentiation period.

## Discussion

One of the most striking consequences of COPD is the reduction in lower limb muscle mass resulting in loss of muscle strength [29,30] which has a significant impact on exercise tolerance [31], quality of life [7] and survival [8]. In the present study we sought to determine the contribution of satellite cell activity in the development of limb muscle atrophy in patients with COPD. Our first assessments were performed directly on muscle specimens using histological characterisation and protein content measurements. Even though the number of satellite cells was similar in all groups of subjects tested, indication of increased regenerative events as evidenced by the increased number of central nuclei, was found in patients with COPD and preserved muscle mass compared to patients with COPD and low muscle mass and healthy subjects. Because satellite cells activation, proliferation and differentiation are strongly regulated by the cell environment, we developed primary cell cultures derived from muscle biopsies to further investigate satellite cells intrinsic capacity during proliferation and differentiation. It was found that satellite cells from patients with COPD have a delay in activation that was followed by a significant reduction in MHC accumulation at the end of the myogenic process demonstrating a defect during myotube maturation. Overall, these results are in agreement with a modification of satellite cells intrinsic capacities that could compromise the regenerative potential in lower limb muscles of patients with COPD.

### Myogenic characteristics of the *vastus lateralis*

#### *Satellite cell number*

In order to sustain an appropriate regenerative function and thus be able to support muscle fiber repair, satellite cell population must remain constant during lifespan. In accordance, the density of satellite cells was similar in the three groups of subjects that were investigated. We and others reported similar results in patients with COPD with or without muscle atrophy [21,32]. Together, these results demonstrate that satellite cell number is maintained in patients with COPD and suggests that chronic lung disease does not have a significant impact on the rate of decline of the satellite cell population [32].

#### *Central nuclei and regenerative events*

Upon activation, satellite cells multiply and commit their development into myoblasts [12]. The capacity of myoblasts to differentiate into myotubes and form new myofibers is the last critical step for muscle regeneration [12]. The number of central myonuclei is an indication of committed satellite cells into the repair process [15,33,34]. Surprisingly we found a higher ratio of central nuclei in patients with COPD without muscle atrophy when compared to patients with COPD and muscle atrophy and healthy subjects, which suggest an increase in the number of regenerative events in this particular group. This observation demonstrates that satellite cells can be activated in this population and also supports the hypothesis that muscle tissue might be more exposed to injury during the progression of the disease [35-37]. On the other hand, in patients with a MTCSA < 70 cm^2^, a decrease in the occurrence of regenerative events compared to patients with COPD and preserved muscle mass is seen, confirming a previous finding by our group [21]. This observation could be interpreted as an exhaustion of the regenerative potential because of satellite cell senescence [21].

#### *MRFs protein levels*

Myogenesis is mainly controlled by four myogenic regulatory factors: MyoD, Myf5, Myogenin and MRF4 which are successively activated upon the initial transcriptional activity of Pax7 by the Notch signaling pathway [38]. MyoD is expressed in muscle satellite cells and mature myofibers [12]. We observed an upregulation of MyoD and Myf5 protein contents in whole muscle extracts from patients with COPD and muscle atrophy suggesting an attempt to accentuate proliferation. These results are in contrast to the study of Plant et al. who did not find any difference in the level of expression of MyoD in whole muscle protein lysates between patients with COPD and healthy subjects [39]. In the group of patients with COPD and preserved muscle mass, the increased expression of Myf5 (proliferation), which is paralleled by a decrease in the expression of Myogenin and MRF4 (differentiation) suggests that the switch between Notch (proliferation) and Wnt (differentiation) signaling pathway is dysfunctional leading to a sustained proliferative state, a finding that is corroborated by a previous study [40]. Furthermore, the increased number of central nuclei observed in this group is in line with the notion that satellite cells are under a sustained proliferative state.

### Myogenic process in satellite cells isolated from *vastus lateralis*

When sufficient muscle tissue was available, we successfully isolated and cultured satellite cells from patients with COPD and healthy subjects. Phenotype was confirmed by positive staining for both Pax7 and MHC, two specific markers only found in muscle cells ensuring the quality of this model [12,41]. Moreover, isolated cells had myoblast-like and myotube-like morphologies and expressed specific MRFs during the proliferation and differentiation steps. This protocol allows the culture of satellite cells isolated from fresh human biopsies with high purity and constitutes a powerful tool to assess dynamic events such as proliferation and differentiation. We found that satellite cells isolated from patients with COPD expressed a delayed activation and compromised differentiation when compared with healthy controls cells.

Interestingly, satellite cell adhesion to culture dish was delayed in patients with COPD, suggesting an impaired response to adhesion signals. Cellular proliferation is an essential event for muscle regeneration because the expansion of myogenic cells is needed to provide a sufficient number of new myonuclei for tissue repair [42]. Thus, satellite cell activation must occur within 6 hours post-injury and be followed by robust proliferation within 48 hours to induce an effective muscle regeneration [43,44]. Although unexplained, the delay in satellite cell adhesion observed in COPD supports the notion that the regenerative process could be altered in this patient population. To further characterize satellite cell activity in the context of COPD, we specifically compared the rate of proliferation of cultured cells isolated from muscle specimens and found that once activated, satellite cells from patients with COPD were able to compensate for their initial delay and produce even more myoblasts after 96 hours in culture than controls.

The proliferation of satellite cells in the context of COPD followed a distinct pattern compared to healthy subjects. Excessive oxidative stress is able to induce a delay in myoblast proliferation [45] and extensive inflammatory response alters differentiation. Because oxidative stress and low grade of systemic inflammation have been reported in COPD [46-48], this ambient milieu could promote dysfunctional proliferation pattern of satellite cells in COPD. Our conditions for cell culture were optimal and withdrawal of inflammatory and/or oxidative stress stigmas might explain the recrudescence in the proliferation rate seen after a few days in the culture dish.

#### *Expression of myogenic regulatory factors during myogenesis*

The basic–helix-loop-helix (bHLH) MRFs Myf5, MyoD, myogenin, and MRF4 act as transcriptional activators of skeletal muscle genes [49]. Myf5 and MyoD act together on a genetic pathway upstream of myogenin and MRF4 to program myogenesis in skeletal muscle cells and it has been suggested that they can counterweigh their own individual actions. For instance, mice lacking functional MyoD gene were found to express about four-fold higher levels of Myf5 leaving them without abnormalities in skeletal muscle development [50]. In our study, during the process of differentiation of satellite cells, we found a three-fold increase in Myf5 content in COPD even if MyoD accumulation was similar between both groups. This observation suggests that MyoD activity might be compromised in satellite cells growing in a distinct environment such as in COPD. Because Myf5 alone is able to support the appropriate progression of myogenesis as demonstrated by the similar expression of myogenin and MRF4 in both groups in the differentiation phase, we believe that in optimal culture conditions, satellite cells isolated from patients with COPD are able to coordinate their progression toward the formation of myoblasts.

However, the last step of differentiation appears to be affected in COPD. Indeed, the reduced MHC protein accumulation observed in satellite cells from patients with COPD is a strong evidence of an altered differentiation capacity. It can be speculated that the inability of myoblasts to fuse together, a prerequisite step to engage the late MRFs into their transcriptional activity, could explain this finding [51,52]. Altered transitional signaling in the Notch/Wnt balance is a likely explanation for this observation. The novel finding that impaired Notch signaling may be partly responsible for the loss of myogenic potential in aged muscle is intriguing, and provides a potential clue into the mechanisms underlying this altered regenerative process [34,53]. Because this study was not designed to specifically address the function of these membrane proteins, further experimentations are warranted to better characterize their role in the regenerative activity of satellite cells isolated from patients with COPD.

### Limitations of the study

Because of the nature of the study design, it is difficult to determine causality between impaired regenerative process and muscle atrophy development in COPD. A small number of muscle specimens were used for cell isolation and culture. As a consequence, it was not possible to test the impact of muscle atrophy on satellite cell behaviour when placed into culture. In a similar way, the study was not designed to pursue mechanistic investigations. Even if these limitations are present, this innovative study brings some exciting results that provide foundation for further experimentations aimed at understanding the impact of impaired muscle regeneration on the atrophying process observed in COPD and other chronic conditions.

## Conclusion

We provide strong evidence of a defect in satellite cells biology in the *vastus lateralis* muscle of patients with COPD. Because cultured satellite cells isolated from muscle samples obtained in patients with COPD have atypical expression pattern of MRFs that may result in poor MHC accumulation in myotubes compared to healthy subjects, we believe that muscle tissue regeneration could be impaired in COPD. The relevance of this finding for the development of muscle atrophy in this disease will need to be considered in future studies. Further efforts to delineate how the *in vivo* environment (growth factor availability, nature and intensity of the stressors) can affect the intrinsic capacity of satellite cells to regenerate muscle tissue will be useful.

## Competing interests

The authors declare that they have no competing interests.

## Authors’ contribution

All authors participate in the study conception, M-E.T. perform cell culture, protein analysis, statistical analysis and draft the manuscript; M-È.P. help with immunostaining and ensure ethic approval; B.L. realised clinical data acquisition; F.M. perform muscle biopsies and R. D. supervise data analysis and statistical analysis. All authors read and approved the final manuscript.
